# The Effect of Vaccines on Older African Americans Emotional Experiences During the COVID-19 Pandemic

**DOI:** 10.1093/geroni/igab046.2695

**Published:** 2021-12-17

**Authors:** Sophie Hanna, Dwana Bass, Sarah Shair, Loraine DiCerbo, Bruno Giordani, Voyko Kavcic

**Affiliations:** 1 Wayne State University, Detroit, Michigan, United States; 2 Institute of Gerontology - Wayne State University, Sterling Heights, Michigan, United States; 3 University of Michigan, University of Michigan, Michigan, United States; 4 Neuropsychology/Michigan Alzheimer's Disease Research Center, University of Michigan, Ann Arbor, Michigan, United States

## Abstract

The COVID-19 pandemic is an unprecedented health emergency that has forced a change in the daily life of all individuals across the nation for over a year. As vaccinations have begun in Detroit, we examined their effect on older African Americans’ emotional experiences and intent to get vaccinated during the pandemic to help understand how persons make decisions to accept vaccinations. For this study, 194 community-dwelling older African Americans (mean age = 75, age range = 64-94) were recruited from the Wayne State Institute of Gerontology Healthier Black Elders Center and general Detroit area. A telephone survey was administered to assess pandemic experience including demographics, emotional responses (e.g., gratitude, happiness, anger, fear), everyday stressors (e.g., economic problems, reduced privacy), and vaccination attitude (e.g., concern over safety, intent to vaccinate). Of the 194 participants, 149 completed the survey before the first vaccination occurred in the United States on December 15, 2020, and 45 completed the survey after. Participants had not yet been vaccinated, but 67% said they would as soon as available. Participants in the post-vaccination group, as compared to pre-vaccination group, showed increases in stress-related locus of control (p=.03) and reported being more likely to get vaccinated (p=.02). They showed decreased worry about availability of health and safety supplies (p=.01), reduced perceived stress (p=.02), and a decrease in fears of COVID-19 (p=.05) and vaccination safety (p<.001). The current study highlights the effect of vaccinations on the attitudes and emotions experienced by an older minority population living in an urban area.

